# Dietary vitamin E: effect on oxidative stress, maze learning performance, and anxiety behaviors in rats

**DOI:** 10.1093/tas/txaf049

**Published:** 2025-05-21

**Authors:** Cayla J Iske, Anna K Johnson, Kelly L Kappen, Roni M Deever, Cheryl L Morris

**Affiliations:** Department of Animal Science, Iowa State University, Ames, IA, USA; Omaha’s Henry Doorly Zoo and Aquarium, Omaha, NE, USA; Department of Animal Science, Iowa State University, Ames, IA, USA; Omaha’s Henry Doorly Zoo and Aquarium, Omaha, NE, USA; Omaha’s Henry Doorly Zoo and Aquarium, Omaha, NE, USA; Department of Animal Science, Iowa State University, Ames, IA, USA; Omaha’s Henry Doorly Zoo and Aquarium, Omaha, NE, USA

**Keywords:** behavior, Long Evans rat, radial-arm maze, welfare, rodent

## Abstract

The brain is particularly susceptible to oxidative stress (OS) and damage to membranes is associated with learning and memory decline, impacting animal welfare. Vitamin E is an antioxidant which crosses the blood-brain barrier. Our objectives were to assess the impact of dietary vitamin E concentrations (20, 90, and 400-ppm) on markers of OS, maze learning performance (MLP), and anxious behaviors in 3-wk old Long-Evans rats. Vitamin E concentrations, antioxidant enzymes (superoxide dismutase [SOD] and glutathione peroxidase [GPx]), and oxidative protein damage (protein carbonyls [PC]) were measured in plasma or serum. Lipid damage (thiobarbituric acid reactive substances [TBARS]) was measured in serum and hippocampus. Anxious behaviors, including freezing and grooming, and MLP were assessed in an eight-arm radial maze over 5 weeks. Activity of SOD was lower (*P* = 0.002), and PC concentrations were higher (*P* = 0.022) in the 400-ppm group (1.0 U/mL; 0.7 nmol/mg) compared to the 20 (2.9 U/mL; 0.5 nmol/mg) and 90 (1.7 U/mL; 0.5 nmol/mg). Plasma vitamin E increased (*P* < 0.050) with dietary treatment and SOD decreased as plasma vitamin E increased (R^2^ = 0.46; *P* = 0.002) but PC (R^2^ = 0.16; *P* = 0.090) concentrations tended to increase with plasma vitamin E. Dietary treatment did not impact (*P* > 0.050) maze learning performance. Rats fed 20 ppm vitamin E exhibited greater freezing frequency and duration (*P* < 0.001) compared to other treatment groups, indicating heightened anxiety. The 400-ppm group exhibited lowest grooming frequency and duration (*P* < 0.001), possibly indicating less anxiousness. Working memory errors increased with serum TBARS concentrations (R^2^ = 0.26; *P* = 0.033). In conclusion, higher dietary vitamin E concentrations reduced anxious behaviors, but did not alter MLP and was correlated with increased OS. These results suggest high concentrations of dietary vitamin E are not beneficial for rat welfare.

## Teaser

More of a good thing is always better, right? Not necessarily. This work shows there might be a limit to the benefits of dietary antioxidants.

## INTRODUCTION

Oxidative stress (OS) is defined as an imbalance of pro- and anti-oxidant compounds in favor of pro-oxidants (Sies, 1985). Pro-oxidants, namely reactive oxygen species (ROS), are highly reactive due to an unpaired electron which can attack and damage biological molecules including proteins, lipids, DNA, and RNA (Halliwell and Gutteridge, 2015). Due to its high polyunsaturated fatty acid (PUFA) and iron content along with high oxygen consumption (Halliwell, 2001), the brain is particularly susceptible to OS. Brain damage from OS can result in degeneration (Fukui et al., 2002), alteration of neurotransmitters and their receptors (Joseph et al., 1998) and compromised neuronal excitability or synaptic function (Wu et al., 2010). Many neurodegenerative diseases such as Parkinson’s and Alzheimer’s disease (Niedzielska et al., 2016) as well as a general decline in learning, memory (Revel et al., 2015), and anxiety (Bouayed et al., 2009) have been linked to OS.

Nutrition plays a key role in OS through the provision of non-enzymatic and dietary antioxidants. Antioxidant supplementation may improve learning performance in species such as humans (Morris et al., 2002), dogs (Milgram et al., 2002), and rodents (Joseph et al., 1998; Fukui et al., 2002; Zaidi and Banu, 2004; Wu et al., 2010) and has also been shown to mitigate anxious behaviors in mice and rats (Xu et al., 2014). Vitamin E is an effective antioxidant in modulating OS, particularly in the brain (Wu et al., 2010; Gropper and Smith, 2013). However, published rodent studies have not considered specific and measured dietary vitamin E concentrations with OS and resulting learning performance or anxious behaviors (Joseph et al., 1998; Fukui et al., 2002; Zaidi and Banu, 2004; Wu et al., 2010). Rodents can serve as models for other species making results applicable to exotic, pet, and laboratory rodents. Therefore, the objectives of this study were to assess the impact of three dietary vitamin E concentrations in rat (1) OS markers and (2) learning performance and anxiety behaviors in a radial arm maze (RAM). It was hypothesized that rats fed higher dietary vitamin E concentrations would have lower levels of OS markers, superior learning performance and fewer anxiety behaviors.

## METHODS

### Animals, Housing, and Testing

Throughout the entire study, rats were housed at 24 ± 2 °C with 60% relative humidity (RH) in their home cages. Lighting was on a 10-h light:14-h dark cycle with 27.4 average LUX.

### Experimental Procedure

#### Infant period.

Eighteen, 3-wk-old Long-Evans rats were obtained from two litters born from the feeder animal colony at Omaha’s Henry Doorly Zoo & Aquarium (9 males and 9 females; initial body weight [BW] 29.1 to 45.9 g). Young rats were housed individually in standalone plastic cages that measured 46 H x 24 W x 20 L cm. Each cage had wire lid. Cages contained approximately 2.5 cm of corncob bedding and one 10 W x 20 L cm piece of cardboard, which was replaced bi-weekly. Water was provided ad libitum via plastic 550 mL water bottles (Girton Manufacturing Co., Inc., Millville, PA. Model 16 - 38).

#### Baseline period.

*Wk 1*. All rats were fed every morning (ad libitum) a 17-ppm vitamin E (dl-α tocopheryl acetate) diet (TestDiet^®^, St. Louis, MO) in their home cage. Feed intake was measured daily (0.1 g; Mettler-Toledo, LLC., Model XP8001M, Columbus, OH). Study rats were too small for blood collection without compromising long-term health. Therefore, blood was collected from littermates (one male and female from each litter) that were not enrolled in the study. At the conclusion of the baseline period, littermate rats were anesthetized by veterinary staff in an induction chamber with sevoflurane (SevoFlo^®^, Zoetis Inc., Kalamazoo, MI; 8% in 1L oxygen). Once anesthetized, littermate rats were maintained on sevoflurane (4% in 1L oxygen) via facemask, while veterinary staff collected 2 to 3 mL of arterial blood via cardiac puncture using a 22-gauge needle, 3 mL syringe (Monoject™, Covidien™, Minneapolis, MN) to exsanguination and heartbeat was absent. Blood was collected into one Vacutainer tube (Becton, Dickinson and Company, Franklin Lakes, NJ) containing 1.8 mg EDTA/mL for plasma collection and centrifuged at 1000 x g for 10 min to separate plasma which was stored at -80 °C.

#### Dietary acclimation period.

*Wks 2 - 5*. Rats were blocked into three groups and fed their assigned vitamin E dietary treatments (TestDiet^®^, St. Louis, MO; 100% ad libitum) in the morning in their home cage. Utilizing a complete randomized block design, study rats were blocked by sex, litter, and BW and were randomly assigned to one of three dietary treatment groups containing increasing concentrations of dl- α tocopheryl acetate: 1) 20-ppm: BW 72.7 g ± 15.0 (*n* = 6); 2) 90-ppm: BW 73.4 g ± 10.8 (*n* = 6); or 3) 400-ppm: BW 73.2 g ± 6.8 (*n* = 6).

All diets were formulated to meet nutrient growing rat requirements (National Research Council, 1995) apart from, and varying only in, vitamin E. Diets were stored refrigerated at 2 °C and 35% RH. The vitamin E requirement for rats is 18-ppm (27 IU/kg) and many commercially available rat diets are formulated at approximately 50-ppm (Mazuri^®^ [PMI Nutrition International], St. Louis, MO) Rat & Mouse Diet (5663); LabDiet^®^ Laboratory Rodent Diet (5001); LabDiet^®^ Rat Diet (5012) (Land O’Lakes, Inc., St. Louis, MO). Following formulation, experimental diets were analyzed (HPLC quantification: AOAC Method 971.30 AOAC, 2011) and contained 22.0, 87.5, and 388.8-ppm of vitamin E, respectively. These dietary treatments will be referred to as 20, 90, and 400-ppm. Rats were maintained on their assigned dietary treatments throughout the study (wks. 2 - 11). Feed intake was measured daily (0.1 g; Mettler-Toledo, LLC., Model XP8001M, Columbus, OH). During wk 5, rats were handled daily for five consecutive min by one of four research staff for habituation. Handling included physical contact with rats in their cage, picking up rats by supporting under the abdomen, and holding each individual rat unrestrained and allowing each rat to walk on their handlers.

#### Exploratory period.

**Figure 1.**
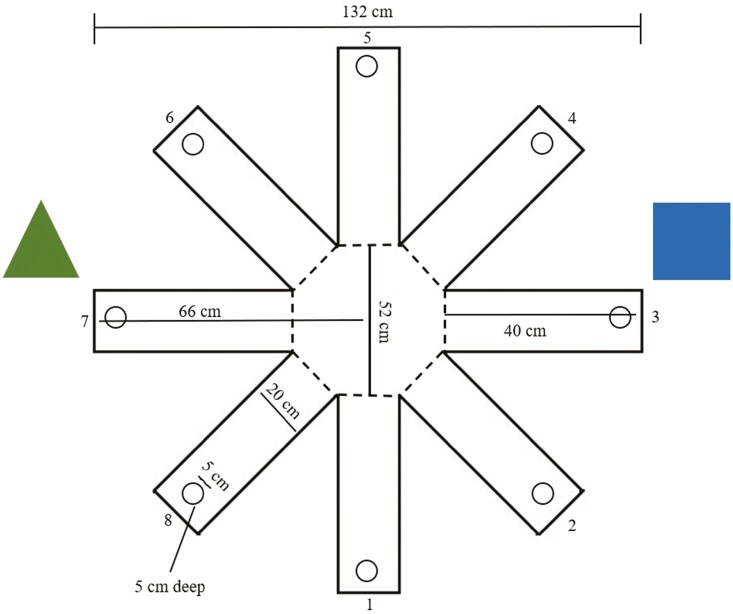
Schematic representation of the eight-arm radial maze used for maze learning performance and rat anxiety behaviors for Long Evans rats fed diets containing 20, 90 or 400 ppm of vitamin E (dl-α tocopherol) for the exploratory period (wk 5) and nine wks of testing (wk 6 to 11). Rats were tested for 10-min on six consecutive days over five consecutive wks. The RAM was elevated 10 cm above the floor on a wooden platform (114 cm diameter). The maze was 132 cm in diameter with white, opaque Plexiglas floor and clear Plexiglas walls (40 cm height). Each radial arm was 20-cm wide and 40-cm long radiating from an octagonal center (52 cm diameter). A white guillotine-style door (20.5 × 40 cm) was located between each arm and the maze center. Centered at the end of each arm was a clear Plexiglas food cup 5 cm in diameter and depth, recessed 5 cm below the maze floor. The maze remained in a constant orientation throughout the experiment. Black fabric curtains were hung on shelving units (216 × 264 cm) and blocked all visual cues except the room door, ceiling, and two, fixed extra-maze cues for learning landmarks. One cue was a blue paper square (30.5 L x 30.5 W cm) located at the end of arm number 3 and one green paper triangle (30 × 30 cm) located at the end of arm 7. Shapes were hung on the curtains above the top of maze walls (32 cm above the floor). The maze was located in a 2.72 L x 2.64 W m 50 LUX room maintained at 22 ± 2 °C with 57% RH.

*Wk 6*. Rats were individually carried approximately 6 m in their home cages from the colony room to the maze testing room. Individual rats were removed from the home cage and placed into the center of an eight-arm radial maze facing arm 5 (RAM; Country Plastics, Ames, IA; Figure 1). The RAM was elevated 10 cm above the floor on a wooden platform (114 cm diameter). The maze was 132 cm in diameter with white, opaque Plexiglas floor and clear Plexiglas walls (40 cm height). Each radial arm was 20-cm wide and 40-cm long radiating from an octagonal center (52 cm diameter). A white guillotine-style door (20.5 × 40 cm) was located between each arm and the maze center. Centered at the end of each arm was a clear Plexiglas food cup 5 cm in diameter and depth, recessed 5 cm below the maze floor. The maze remained in a constant orientation throughout the experiment. Black fabric curtains were hung on shelving units (216 × 264 cm) and blocked all visual cues except the room door, ceiling, and two, fixed extra-maze cues for learning landmarks. One cue was a blue paper square (30.5 L x 30.5 W cm) located at the end of arm number 3 and one green paper triangle (30 × 30 cm) located at the end of arm 7. Shapes were hung on the curtains above the top of maze walls (32 cm above the floor). The maze was located in a 2.72 L x 2.64 W m 50 LUX room maintained at 22 ± 2 °C with 57% RH.

Rats explored the RAM for 10 min without data collection for 6 consecutive days. Rat exploratory testing order remained consistent, alternating between dietary treatment groups. Exploratory session began at 08:00. Eight apple pieces, one piece per arm, (without skin weighing ~1.5 g total) were placed within 12 cm of the maze center on the first exploratory day and moved 10 - 15 cm closer to the cups at the end of the maze arms on each successive exploration day. On the last day of the exploratory period, all apple pieces were placed in respective recessed food cups. Rats continued to be fed at 100% ad libitum of their treatment diet daily in their home cage immediately after they had completed their individual exploratory period testing.

#### Maze learning performance and anxiety behavior testing period. Wks. 7 - 11.

Maze testing wk 1 was wk 7 of the entire study, and testing wk 5 was wk 11 of the entire study. Rats were individually tested in the RAM for maze learning performance and rat anxiety behaviors. Maze learning performance and anxiety behavior testing occurred in the same order from the exploratory period and consisted of two daily trials with a three-hour separation between trial one and two. Rats were transported in their home cages to the maze, removed from their home cage and placed in the maze center, facing arm 5. In both trials, one apple piece (without skin weighing ~25 mg) was accessible in four randomly assigned (via random number generator) maze arms, defined as “goal arms.” The goal arms remained consistent for all rats throughout maze learning performance and anxiety behavior testing. To avoid scent bias, a mesh barrier was inserted above one apple piece in food cups (without skin weighing ~25 mg) in food cups of the remaining four arms (defined as “non-goal arms”) to block rat access. In the first trial, guillotine doors blocked access to non-goal arms. During the second trial, guillotine doors were removed and rats had access to all eight RAM arms.

Rats were returned to their home cage after they had consumed food in all goal arms or 10 min had elapsed. The maze was thoroughly cleaned with 70% ethanol between all trials to prevent odor cues. All uneaten apple pieces were placed in the rat’s home cage at the end of each testing period. Maze learning performance and anxiety behavior testing was conducted for six consecutive days each wk, or until the rat reached criterion which was defined as making no error in the second trial for two consecutive days.

In all trials, one experimenter was present in the maze room during testing and was visible to each rat. Learning performance measures included latency to find all apple slices (TTF; seconds), latency to consume all apple slices (TTC; seconds), entry into a non-goal arm (long term or reference memory errors [RME] frequency) or reentry into an arm after the food had already been consumed (short term or working memory errors [WME] frequency) which were collected using continuous live observation. A total errors (TE) category was created by summing RME + WME (frequency). Anxiety behaviors included urination, defecation, and vocalization frequency were also collected utilizing a continuous live observation methodology (Table 1).

**Table 1.** Ethogram for long evans rats in an eight-arm radial maze^1^

Measure	Unit	Description
***Maze Learning Performance*** ^2^		
Time to find food (TTF)	Seconds	Latency from rat being placed in maze center until nose broke horizontal plane of recessed food cup with accessible apple slices (goal arms)
Time to consume food (TTC)		Latency from rat being placed in the maze center until rat consumes all accessible apple slices (goal arms)
Reference memory errors (RME)	Frequency^4^	Frequency rat placed both front feet in a maze arm that did not contain accessible apple slices (non-goal arm)
Working memory errors (WME)		Frequency rat placed both front feet in a maze arm in which the rat had already consumed the apple slice
Total errors (TE)		Frequency rat placed both front feet in a maze arm which did not contain an accessible apple slice or an arm in which the rat had already consumed the apple slice
***Rat Anxiety Behaviors*** ^3^		
Rear	Frequency and duration^5^	Rat stood on its hind legs with its back straight
Freeze		Rat was immobile on all four legs with no body movement for at least 3 seconds
Groom		Rat licked body or paws and rubbed them on its body
Urination	Frequency	Rat excreted urine in maze which was visible to the human live maze observer
Defecation		Rat excreted feces in maze which was visible to the human live maze observations
Vocalization		Rat emitted a vocal frequency detectable to the human live maze observer

^1^Ethogram adapted from (Rousseau et al., 2000). Rats were tested for 10-min on six consecutive days over five consecutive wks.

^2^Maze performance parameters were collected by one observer during live maze testing.

^3^Anxiety behaviors rear, freeze, and groom were collected by one observer during video recording observations. Urination, defecation, and vocalization were recorded during live maze testing.

^4^Frequency defined as average number of occurrences per maze trial.

^5^Duration defined as average number of seconds each occurrence lasted.

A video camera (Sony HandyCam, HDR-SR7, Tokyo, Japan) was positioned 3.4 m above the ground in the maze room using an articulated arm mount attached to a ceiling support beam. Video continuously recorded the second trial in color at 30 fps. Continuous behavioral observations were conducted by one observer masked to dietary treatments. The observer was trained by a trainer with previous behavioral observation experience as previously described by Caro et al. (1979) and Martin and Bateson (1993). Inter-observer reliability testing was conducted using three maze videos that included one rat per dietary treatment, in trial two, of study wk 7, and inter-observer agreement was ≥ 93% (Caro et al., 1979). Frequency (occurrences/trial) and duration (seconds/occurrence) for rear, freeze, and groom behaviors were recorded (Table 1). Rats were fed at 80% ad libitum of their treatment diet daily in their home cage immediately after they had completed their individual maze learning performance and anxiety behavior testing period.

### Postmortem

After meeting maze criteria (no errors for two consecutive days), or after 5 wks of maze training during the Maze Learning Performance and Anxiety Behavior Testing Period, rats were fasted overnight and anesthetized by veterinary staff in an induction chamber with sevoflurane (SevoFlo^®^, Zoetis Inc., Kalamazoo, MI; 8% in 1L oxygen) and weighed to the nearest 0.1 g (Adam Equipment^®^, Compact Balance, model 1001, Oxford, CT). Once anesthetized, rats were maintained on sevoflurane (4% in 1L oxygen) via facemask and 6.5 to 16.0 mL of arterial blood was collected via cardiac puncture (22-gauge needle, 3 mL syringe; Monoject™, Covidien™, Minneapolis, MN) to exsanguination and heartbeat was absent. Blood was divided into two separate Vacutainer tubes (Becton, Dickinson and Company, Franklin Lakes, NJ), one serum separator tube and one tube containing 1.8 mg EDTA/mL for plasma collection. Blood was centrifuged at 1000 x g for 10 min to separate serum and plasma and stored at -80 °C until analyses. The hippocampus was also collected and approximately 25 mg of tissue was homogenized with 250 µL of RIPA buffer and centrifuged at 1600 x g for 10 min. Supernatant was collected and stored at -80 °C until analyses.

### Oxidative Stress Markers

Multiple markers of OS were measured in plasma and serum. Analyses were conducted using commercially available assay kits purchased (Cayman Chemical Company, Ann Arbor, MI) and were performed according to the recommendations of the manufacturer and analyzed via microplate spectrophotometer (Epoch, BioTek Instruments Inc., Winooski, VT). Protein carbonyl (PC) (catalog number 10005020) concentrations were measured as an indicator of protein damage in plasma and data were reported as nmol/mg protein. Protein content of blood plasma was determined via bovine serum albumin assay (Smith et al., 1985) using the Pierce™ BCA Protein Assay Kit (Thermo Scientific, catalog number: 23227, Waltham, MA). Thiobarbituric acid reactive substances (TBARS) (catalog number 700870) were measured as indicators of lipid damage in serum and hippocampus. No dilutions were necessary in serum and data were reported as µM of malondialdehyde (MDA). Antioxidant enzymes superoxide dismutase (SOD) and glutathione peroxidase (GPx) are two of the most commonly measured markers to evaluate OS. Both SOD and GPx (catalog numbers 706002 and 703102, respectively) were measured in plasma. Samples were diluted 1:4 in sample buffer before being assayed for SOD activity and were expressed as Unit/mL (one unit defined as the amount of enzyme that resulted in 50% dismutation of the superoxide radical). Activity of GPx was measured in samples diluted 1:20 with sample buffer and was expressed as nmol/min/mL. All assays were run in triplicate in a 96-well plate with the exception of PC which is analyzed in duplicate.

Plasma samples also were sent to Arizona State University for vitamin E (d1-α tocopherol acetate) analyses. Vitamin E was analyzed via reverse-phase HPLC as previously described (McGraw et al., 2006) using an Agilent 1100 Series (Santa Clara, CA).

### Statistics

The home cage containing one rat was the experimental unit. All statistical analyses were conducted using SAS^®^ (SAS Inst. Inc., Cary, NC). Final body and hippocampus weights and OS markers were normally distributed; therefore, they were analyzed using the Mixed Models procedure. Diet, litter, and sex were tested as fixed effects and initial body weight was tested as a covariate. Daily diet intake was averaged within dietary treatment group and assessed using the Mixed Models procedure. Plasma vitamin E concentrations were also normally distributed and analyzed in the Mixed Models procedure in the same way with the additional assessment of sex and littermate baseline vitamin concentrations as a covariate.

Rat maze learning performance data (TTF, TTC, RME, WME and TE) were non-normally distributed and analyzed in PROC GLIMMIX using a gamma distribution. Rat was included as a random effect. Rat anxiety behaviors (rear, freeze and groom) frequency and duration data also were non-normally distributed and analyzed in PROC GLIMMIX using a gamma distribution, but could not be analyzed using a random statement. The statistical model included diet, litter, sex, and maze testing wk (1 to 5; Maze Learning Performance and Anxiety Behavior Testing period ([wks 7 to 11 of study]) as well as a diet × testing wk interaction. Urination frequency was analyzed in PROC GLIMMIX using a gamma distribution and a random effect of rat. Defecation and vocalization frequencies were analyzed with a binomial distribution without a random statement due to lack of conversion, likely due to low occurrence. The I-Link option was used to transform the LS mean and standard error values back to the original measurement units. A *P*-value of ≤ 0.050 was considered significant. When the fixed effect was a significant source of variation, different levels within the fixed effect were separated using the PDIFF option in SAS. Multiple regression analysis was conducted in PROC REG to assess for correlations between plasma vitamin E, OS markers, average maze performance parameters, and behaviors.

## RESULTS

### Animals and Diets

Baseline plasma vitamin E concentrations for littermates (not used in the study) were: litter 1 males, 0.9 and 2.4; litter 1 females, 1.0 and 2.5; litter 2 males, 1.2 and 2.5; and litter 2 females, 0.9 and 2.1 µg/mL. Baseline vitamin concentrations were not significant (*P* ≥ 0.559) covariates in statistical analyses of final plasma vitamin concentrations.

Rats on the 20-ppm dietary treatment consumed less feed per day (16.4 ± 0.3 g as-fed) than rats fed 90-ppm (18.2 ± 0.3 g) and 400-ppm (17.7 ± 0.3 g) respectively (*P* < 0.001). Body weights between dietary treatments over the study did not differ (*P* = 0.472). Final body weights did not differ (290.6 ± 14.9 [20-ppm], 313.7 ± 14.9 [90-ppm] 304.1 ± 14.9 g [400-ppm]; *P* = 0.564). In addition, hippocampus weights were similar between dietary treatments (0.06 ± 0.01 g [20-ppm], 0.06 ± 0.01 g [90-ppm] and, 0.06 ± 0.01 g [400-ppm]; *P* = 0.892).

### Oxidative Stress Markers

As dietary vitamin E increased from 20 to 400 ppm, SOD activity decreased (*P* < 0.001). This was in contrast to the 40% increase in PC concentration (*P* = 0.004) between rats fed 20-ppm and 400-ppm. No differences were detected between rats on dietary treatments for plasma GPx (*P* = 0.720). Protein carbonyl (PC) concentrations were significantly higher (*P* = 0.024) in rats fed the 400-ppm dietary treatment (0.7 nmol/mg) compared to those fed the 20-ppm dietary treatment (0.5 nmol/mg) with no other differences detected for PC. Intra- and inter-assay CV’s for PC were 4 10.0 and 29.3%, respectively (Table 2).

**Table 2.** Oxidative stress markers and vitamin E concentrations in blood and hippocampus TBARS of Long Evans rats fed diets containing 20, 90, and 400 ppm vitamin E (α-tocopherol)^1^

Measure	Dietary Vitamin E, ppm			SEM	*P*-Value
	20	90	400		
SOD, U/mL^2^^,^^3^	2.9^a^	1.7^b^	1.0^c^	0.2	< 0.001
GPx, nmol/min/mL	1939.9	1660.6	1919.5	374.2	0.720
Serum TBARS, µM MDA	12.6	11.5	12.3	1.6	0.767
Hippocampus TBARS, µM MDA	18.4	19.5	20.2	2.3	0.743
Vitamin E, µg/mL	1.2^a^	2.2^b^	3.5^c^	0.5	0.001
PC, nmol/mg	0.5^a^	0.5^a,b^	0.7^b^	0.1	0.004

SOD, superoxide dismutase; GPx, glutathione peroxidase; TBARS, thiobarbituric acid reactive substances; PC, protein carbonyl; SEM, standard error of mean.

^a-c^Means within a row lacking a common superscript letter are different (*P* < 0.050). Rows with no superscript did not differ statistically (*P* > 0.100).

^1^SOD, GPx, and PC are measured in plasma. Rats fed respective diets for nine wks.

^2^One unit of SOD is defined as the amount of enzyme that resulted in 50% dismutation of the superoxide radical.

^3^Assay values fell outside the range of the standard curve.

**Figure 2.**
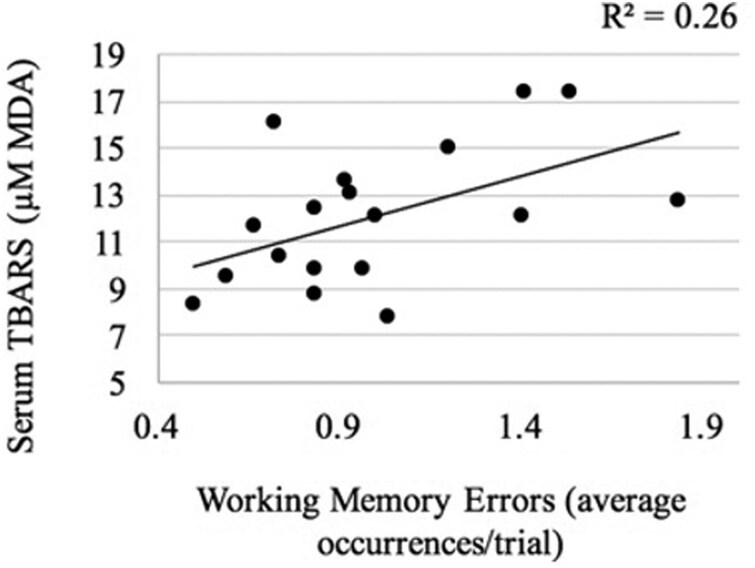
Linear regression of serum lipid peroxidation (TBARS) and working memory errors (WME) (*P* = 0.033) committed by rats in a radial arm maze.

No treatment differences were detected for serum or hippocampus TBARS (*P* ≥ 0.743). Intra- and inter-assay CV’s for SOD, GPx, serum- and hippocampus TBARS were 4.9 and 45.4, 6.8 and 31.8, 4.3 and 24.2, 3.1 and 19.7, respectively. Final plasma vitamin E concentrations increased (*P* = 0.001) with dietary vitamin E (1.2, 2.2 and 3.5 µg/mL for 20, 90 and 400-ppm, respectively; Table 2). As dietary vitamin E increased, activity of SOD decreased (*P* = 0.002; (R^2^ = 0.46)) and PC concentrations (R^2^ = 0.16; *P* = 0.090) tended to increase. When regression analysis was conducted with OS markers and average maze performance, serum TBARS concentrations increased (R^2^ = 0.26; *P* = 0.033) with WME (Figure 2). Markers of OS were not correlated with maze learning performance or anxiety behaviors.

## MAZE LEARNING PERFORMANCE AND ANXIETY BEHAVIOR TESTING PERIOD

### Diet × Test Wk

**Figure 3.**
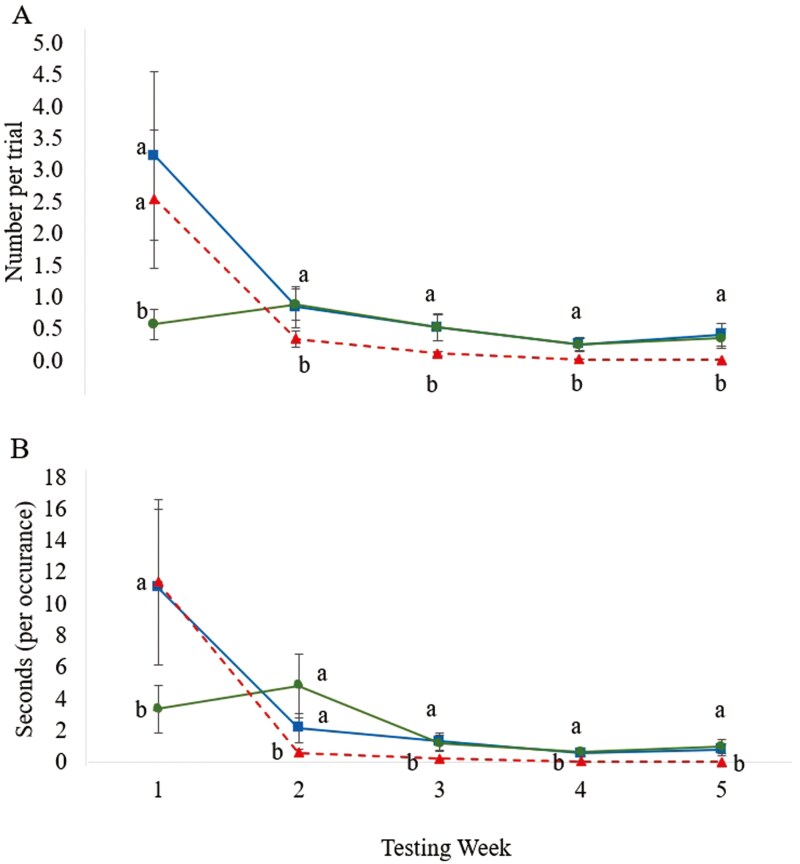
Long Evan rat grooming frequency (A) and duration (B) behaviors when fed diets containing 20 (*blue square*), 90 (*green circle*), and 400 (*red triangle*) ppm vitamin E in an eight-arm radial maze^1,2^(*P* = 0.003). Data points lacking a common superscript letter are different (*P* < 0.050). ^a^ Maze testing wks 1 to 5 are during the maze learning performance and anxiety behavior testing period (wks 7 to 11 of study). ^1^ Rats were fed test diets for nine weeks and maze testing was conducted over the last five weeks of the study for 10-min on six consecutive days via live observations using a preset ethogram and continuous behavioral sampling. ^2^ Maze: 132 cm in diameter with white, opaque Plexiglas floor and clear Plexiglas walls (40 cm height). Each radial arm was 20-cm wide and 40-cm long radiating from an octagonal center (52 cm diameter). A white guillotine-style door (20.5 × 40 cm) was located between each arm and the maze center. Centered at the end of each arm was a clear Plexiglas food cup 5 cm in diameter and depth, recessed 5 cm below the maze floor.

Diet × test wk interactions were not observed to be different for rat maze learning performance measures (*P* ≥ 0.050). Grooming frequency increased from wk 1 (0.6 occurrences/trial) to wk 2 (0.9 occurrences/trial) for rats fed 90-ppm but decreased across testing wks 2 through 5 for all dietary treatments (*P* = 0. 003; Figure 3A). Grooming duration for rats fed 90-ppm vitamin E (3.3 sec/occurrence) was 71% shorter (*P* = 0.049) than for rats fed the 400-ppm (11.4 sec/occurrence) dietary vitamin E in wk 1. However, grooming duration was 88% shorter (*P* = 0.005) in rats fed 400-ppm (0.6 sec/occurrence) compared to those fed the 90-ppm (4.8 sec/occurrence) vitamin E in wk 2. In wks 3, 4, and 5, grooming duration was 85, 95, and 99% less (*P* < 0.050) in 400 ppm compared to 20 and 90-ppm (Figure 3B).

### Diet

There were no differences between rats fed dietary vitamin E treatments for any maze learning performance measures (*P* ≥ 0.118; Table 3) or urination, defecation, vocalization, or rearing (*P* ≥ 0.341; Table 4). There were observed differences for freeze and groom (*P* < 0.001; Table 4). Rats fed the 20-ppm vitamin E treatment froze more and for longer compared with those fed the 90 and 400-ppm vitamin E treatments. Rats fed 400-ppm vitamin E groomed less and for shorter periods compared to rats fed the 20 and 90-ppm vitamin E treatments (*P* < 0.001; Table 4).

**Table 3.** Maze learning performance in an eight-arm radial maze^1^ of Long Evans rats fed diets containing 20, 90 or 400 ppm of vitamin E (α-tocopherol)^2^

Measure^3^	Dietary Vitamin E, ppm			SEM	*P*-Value
	20	90	400		
TTF, seconds	63.2	67.8	64.3	10.9	0.931
TTC, seconds	67.6	71.8	68.7	11.5	0.961
RME, frequency^4^	4.0	4.3	4.3	0.4	0.826
WME, frequency	2.3	1.8	1.9	0.2	0.118
TE, frequency	4.6	5.4	5.2	0.5	0.389

^1^Maze: 132 cm in diameter with white, opaque Plexiglas floor and clear Plexiglas walls (40 cm height). Each radial arm was 20-cm wide and 40-cm long radiating from an octagonal center (52 cm diameter). A white guillotine-style door (20.5 × 40 cm) was located between each arm and the maze center. Centered at the end of each arm was a clear Plexiglas food cup 5 cm in diameter and depth, recessed 5 cm below the maze floor.

^2^Rats were fed test diets for nine weeks and maze testing was conducted over the last five weeks of the study for 10-min on six consecutive days via live observations.

^3^TTF, time to find food: latency to find all apple slices; TTC, time to consume food: latency to consume all apple slices; RME, reference memory errors: entry into an unbaited arm (long term memory errors); WME, working memory errors: reentry into an arm after the food had already been consumed (short term memory errors); TE, total errors: RME + WME.

^4^Frequency defined as average number of occurrences per maze trial.

**Table 4.** Frequency^1^ and duration^2^ of anxiety behaviors in Long Evans rats fed diets containing 20, 90 or 400 ppm of vitamin E (α-tocopherol) in an eight-arm radial maze^3^^,^^4^

Behavior	Dietary Vitamin E, ppm			SEM	*P*-Value
	20	90	400		
Rear, frequency	8.7	8.3	8.4	0.8	0.944
Rear, duration	13.9	13.8	13.6	1.4	0.928
Freeze, frequency	0.02^a^	0.01^b^	0.01^b^	0.002	<0.001
Freeze, duration	0.07^a^	0.02^b^	0.02^b^	0.01	<0.001
Groom, frequency	0.7^a^	0.5^a^	0.1^b^	0.2	<0.001
Groom, duration	1.7^a^	1.6^a^	0.2^b^	0.4	<0.001
Urination, frequency	1.1	1.1	1.3	0.1	0.387
Defecation, frequency	0.008	0.008	0.0001	0.0001	0.353
Vocalization, frequency	0.05	0.02	0.02	0.03	0.341

^a,b^Means within a row lacking a common superscript letter are different (*P* < 0.050).

^1^Frequency defined as average number of occurrences per maze trial.

^2^Duration defined as average number of seconds each occurrence lasted.

^3^Rats were fed test diets for nine weeks and maze testing was conducted over the last five weeks of the study for 10-min on six consecutive days via live observations using a preset ethogram and continuous behavioral sampling.

^4^Maze: 132 cm in diameter with white, opaque Plexiglas floor and clear Plexiglas walls (40 cm height). Each radial arm was 20-cm wide and 40-cm long radiating from an octagonal center (52 cm diameter). A white guillotine-style door (20.5 × 40 cm) was located between each arm and the maze center. Centered at the end of each arm was a clear Plexiglas food cup 5 cm in diameter and depth, recessed 5 cm below the maze floor.

### Testing Wk

No differences were detected across testing wks for urination or defecation anxiety (*P* ≥ 0.156) or for vocalization frequency (*P* = 0.089; Table 5). Weekly maze learning performance parameters differed over the 5 testing wks (*P* < 0.001). For all parameters, rats performed higher TTF, TTC, RME, WME and TE during wk 1. These all decreased by wk 2, and then decreased again at wk 3. Between wks 3 and 5 there were no differences in learning performance parameters. Rear, groom, and freeze frequencies and durations were highest wk 1 and then decreased or plateaued as testing progressed (*P* < 0.001; Table 5).

**Table 5.** Weekly maze learning performance and anxiety behavior testing of Long Evans rats fed diets containing 20, 90 or 400 ppm of vitamin E (α-tocopherol) in an eight-arm radial maze^1-3^

	Testing Wk					SEM	P-Value
	1	2	3	4	5		
***Maze Learning Performance Parameter*** ^***4***^							
TTF	146.4^a^	71.8^b^	51.5^c^	46.6^c^	49.7^c^	0.1	< 0.001
TTC	179.9^a^	73.5^b^	52.8^c^	47.9^c^	52.1^c^	0.1	< 0.001
RME	9.2^a^	4.2^b^	3.5^c^	3.3^c^	3.2^c^	0.1	< 0.001
WME	2.9^a^	2.3^b^	1.5^c^	1.9^c^	1.6^c^	0.2	< 0.001
TE	11.3^a^	5.2^b^	4.0^c^	3.8^c^	3.7^c^	0.1	< 0.001
***Anxiety Behavior*** ^***5***^							
Rear Frequency^6^	28.8^a^	8.3^b^	6.4^b,c^	4.9^c^	5.6^c^	1.6	< 0.001
Rear Duration^7^	53.7^a^	13.8^b^	10.6^b,c^	7.5^c^	8.3^c^	0.2	< 0.001
Freeze Frequency	0.4^a^	0.02^b^	0.004^c^	0.002^d^	0.001^d^	0.1	< 0.001
Freeze Duration	4.7^a^	0.10^b^	0.01^c^	0.003^d^	0.002^d^	1.1	< 0.001
Groom Frequency	1.7^a^	0.6^b^	0.3^c^	0.1^d^	0.1^d^	0.6	< 0.001
Groom Duration	7.5^a^	1.8^b^	0.7^c^	0.2^d^	0.2^d^	0.6	< 0.001
Vocalization Frequency	0.1^a^	0.03^a,b^	0.04^a,b^	0.01^b^	0.01^b^	0.03	0.089

TTF, time to find food; TTC, time to consume food; RME, reference memory errors; WME, working memory errors; TE, total errors.

^a-d^Means within a row lacking a common superscript letter are different (*P* < 0.05). Rows with.

no superscript did not differ statistically (*P* > 0.10).

^1^Maze testing wk 1 was wk 7 of the overall study, and testing wk 5 was wk 11.

^2^Rats were fed test diets for nine weeks and maze testing was conducted over the last five weeks of the study for 10-min on six consecutive days via live observations.

^3^Maze: 132 cm in diameter with white, opaque Plexiglas floor and clear Plexiglas walls (40 cm height). Each radial arm was 20-cm wide and 40-cm long radiating from an octagonal center (52 cm diameter). A white guillotine-style door (20.5 × 40 cm) was located between each arm and the maze center. Centered at the end of each arm was a clear Plexiglas food cup 5 cm in diameter and depth, recessed 5 cm below the maze floor.

^4^Maze performance parameters were collected by one observer during live maze testing.

^5^Anxiety behaviors were collected by one observer during video recording observations.

^6^Frequency defined as average number of occurrences per maze trial.

^7^Duration defined as average number of seconds each occurrence lasted.

maze floor.

## DISCUSSION

The objectives of the current study were to assess the impact of three dietary vitamin E concentrations on markers of OS, maze learning performance, and anxiety behaviors in young rats. Oxidative stress has been linked to several aspects of health and welfare including disease (Lykkesfeldt and Svendsen, 2007) and learning decline (Revel et al., 2015), indicating its value as a possible marker of improved welfare status. Vitamin E is a powerful antioxidant that defends against OS and may be protective of the brain to prevent associated cognitive loss (Fukui et al., 2002; Wu et al., 2010; Alzoubi et al., 2013). The vitamin E requirement for growing rats is 18-ppm (27 IU/kg; National Research Council, 1995) with many commercially available rat diets formulated to contain three times this amount.

### Animals & Diets

Whole rat composition did differ by dietary treatment and has been published previously (Iske et al., 2019). Plasma vitamin E concentrations increased as dietary vitamin E increased as more vitamin E was absorbed from the diet, as has been found in previous studies and other species (Machlin and Gabriel, 1982). However, current study values were lower than previously reported for 13 to 25 wks old rodents supplemented with vitamin E (α-tocopheryl acetate) at concentrations of 30 to 55-ppm and 165 to 240-ppm resulting in circulating vitamin E concentrations ranging from 5.4 to 10.9 and 3.1 to 6.4-ppm, respectively (McGee et al., 1990; Terada et al., 2011; Takahashi et al., 2017). These differences could be explained by species and potentially different absorption levels and rates as previous studies used mice or Wistar rats compared to Long-Evans rats in the current study. McGee et al. also administered vitamin E intraperitoneally, opposed to orally, which could also account for observed differences.

### Oxidative Stress Markers

Previous studies have found wide ranges in rat OS markers, with SOD activity, GPx activity, serum TBARS, and PC ranging from 0.4 to 110 U/mL (Bahrami et al., 2016; Bacanlı et al., 2017), 42.5 to 19,700 nmol/min/mL (Anand et al., 2011; Bacanlı et al., 2017), 0.1 to 25 µM (Terada et al., 2011; Bahrami et al., 2016; Mansour et al., 2017), and 0.1 to 7.6 nmol/mg protein (Jana et al., 2002; Husain et al., 2005), respectively. Our values for measured markers of OS fall in line with those previously published for rats. While many studies have reported reduced blood TBARS and PC and increased SOD with vitamin E supplementation (Haque et al., 2006; Wu et al., 2010; Terada et al., 2011), the current study revealed contradictory results with reduced SOD activity and greater PC concentrations in rats fed 400-ppm vitamin E.

Reduction of SOD activity in response to vitamin E supplementation has been previously reported in rats (Abd Hamid et al., 2011) and may be explained by antioxidant sparing effects (Combs Jr., 2012). However, increases of PC detected in rats fed the 400-ppm dietary treatment indicates heightened OS. Though antioxidants such as vitamin E are typically thought to reduce OS (Xu et al., 2014), vitamin E can also have neutral or pro-oxidant effects. A radical (α-tocopheroxyl) is produced through α-tocopherol’s oxidation to neutralize reactive oxygen species (ROS), and this radical requires other antioxidants to reduce it back to the α-tocopherol form (Combs Jr., 2012). It has been proposed that increasing only vitamin E under OS conditions may exacerbate OS via α-tocopheroxyl radical production (Rietjens et al., 2002) and “co-antioxidant” supplementation, such as coenzyme-Q_10_, may have more positive impacts on OS (Mcdonald et al., 2005). Vitamin E has been shown to trigger oxidation (Kontush et al., 1996), though few studies publish these findings in relation to PC. Direct influence of high dietary vitamin E on PC should be further assessed in future studies as should impacts of co-antioxidant supplementation on OS.

The hippocampus appears to be highly prone to OS (Gamoh et al., 2001; Haque et al., 2006) and is also an area of the brain which processes learning and memory (White and McDonald, 2002). Previously reported lipid peroxidation values in rat hippocampus have differed by more than 60-fold (Gamoh et al., 2001; Fukui et al., 2002; Alzoubi et al., 2013) and are similar to results in the current study (average 25 µM; Yuan et al., 2012). Some studies have demonstrated that vitamin E supplementation reduces OS in rat hippocampus (Joseph et al., 1998; Zaidi and Banu, 2004; Wu et al., 2010) while others, in agreeance with the current study, have shown no difference from controls (Alzoubi et al., 2013). The current study indicates higher intakes of dietary vitamin E in diets of rats is not beneficial and may exacerbate OS in young rats that are considered to be in a normal plane of nutrition. This should be further investigated through examination of more refined OS markers such as OS-related genes and in studies of longer duration to assess if results are short-lived or long-term. It would also be valuable to consider comparing measures in both young and old animals.

### Radial Arm Maze

Dietary vitamin E did not influence maze learning performance. These results agree with a previous study in which 5 to 6 wk old adult male Wistar rats fed high-fat high-carbohydrate diets and supplemented with vitamin E (100 ppm dl α-tocopherol) for six wks did not differ in radial arm water maze performance compared to controls (Alzoubi et al., 2013). However, it should be noted that Alzoubi and colleagues (2013) did not report vitamin E concentrations of the control diet.

Our results disagree with other previous studies that observed differences in maze learning performance with vitamin E supplementation; however, many of the reported studies did not report vitamin E source or concentration or used manipulated animals. For example, vitamin E supplemented 3 and 25 mo old male Wistar rats made 33.3% more correct choices compared to vitamin E deficient rats in a RAM (vitamin E concentrations were not reported; Fukui et al., 2002). Similarly, healthy six-mo old male Fischer 344 rats and 7 to 8 wk old male Sprague-Dawley rats with mild traumatic brain injury supplemented with 500 IU vitamin E (acetate, d/l not specified) had improved performance in a Morris Water Maze (MWM) as demonstrated by 28% greater efficiency (Joseph et al., 1998) and 22% shorter maze latency completion times (Wu et al. 2010) compared to controls. Therefore, while our results add to contradictory data presented in previous research, comparisons are difficult when sources or concentrations are not reported. In the current study, increasing concentrations of dietary vitamin E were assessed in healthy young animals without dietary challenge or physical manipulation, which could be a reason why no effect was observed.

Additionally, discrepancies between our results and previous studies may be further explained by differences in maze types evaluated, rat strain utilized, and rat age. It has been previously acknowledged by behavioral scientists that comparing results from different maze type assessments may not be accurate. Although the RAM, MWM, and radial arm water maze all assess learning and memory, results and outcomes are not always directly comparable (Astur et al., 2004). Additionally, rat strains are often selected based on the research objective(s) and rodent model fitness. Gökçek-Saraç et al., (2015) compared four strains of rats and found differences as high as 71% between strains in latency to complete maze, WME, RME, and TE in a RAM. Young and old rats also may be affected by dietary manipulation. Administration of docosahexaenoic acid (DHA) (300-ppm) to 5 and 100-wk-old male Wistar rats resulted in approximately 65% fewer RME in young rats in a RAM, but did not impact WME (Gamoh et al., 1999). Conversely, both RME and WME declined by nearly 75% in old rats (Gamoh et al., 2001). Several studies have demonstrated that vitamin E supplementation improves learning in 24-mo-old rodents (Mcdonald et al., 2005); however, the use of younger rats in the current study that are considered healthy, may have contributed to different results. This could indicate that vitamin E supplementation is more important for learning as animals age or when nutritional status related to vitamin E has not met recommendations.

In the current study, impact of dietary vitamin E and OS on anxiety behaviors also was evaluated. Grooming (Tsoory et al., 2008), freezing (Steimer, 2011), vocalizing (Sánchez, 2003), urinating and defecating (Kalueff and Tuohimaa, 2005) have all been described as behaviors associated with greater anxiety. In contrast, higher occurrences of rearing are associated with less anxiety (Katz et al., 1981). Grooming frequency and duration in our study decreased as vitamin E concentration increased and also decreased throughout the testing weeks. The motivation behind grooming has been debated, some associating it with anxiety and stress (Kalueff and Tuohimaa, 2005) while others find it to be a “de-arousal” mechanism that occurs after a stressful event (Rojas-Carvajal et al., 2018). This debate revolves around the behavioral temporal frequency. If a behavior declines over repeated exposure, it is thought to be a stress-related behavior occurring during habituation (Brenes et al., 2009). A behavior that increases over time may be playing a part in de-arousal after stress has passed (Rojas-Carvajal et al., 2018). In our study, overall patterns of grooming increased between wks 1 and 2 in rats fed the 90 ppm dietary treatment, aligning with the de-arousal hypothesis, while rats fed the 20 and 400 ppm dietary treatments decreased between all wks, in alignment with the stress hypothesis. However, rats fed the 400-ppm vitamin E diet exhibited overall less grooming compared to 20 and 90 ppm-fed rats in wks 2 through 5 which may indicate less stress (Füzesi et al., 2016).

Freezing is a behavioral fear response which serves to avoid detection and unlike rearing and grooming, the freezing behavior is almost always associated with fear or anxiety in a variety of maze tests (Díaz-Morán et al., 2014). In the current study, the rats fed 20-ppm vitamin E froze more frequently and for longer durations than their counterparts consuming 90 or 400-ppm vitamin E. Dietary factors may be influencing anxiety via serotonin and/or gamma amino butyric acid pathways (Gingrich, 2005) in the brain, but this requires further investigation as it relates to vitamin E. Induced vitamin E deficiencies have been reported to increase rodent anxiety behaviors and OS (Terada et al., 2011). However, vitamin E supplementation (up to 500-ppm [α-tocopherol or α-tocopherol acetate]) also has been shown to increase rodent anxiety behaviors (Kolosova et al., 2006). Our study did not induce vitamin E deficiencies but found heightened freezing behaviors in rats fed lowest concentrations of vitamin E (20 ppm). Though further validation in anxiety-focused mazes and tests are required, our results suggest higher dietary vitamin E diets (90 and 400-ppm) may reduce anxious behaviors as seen in reduced grooming and freezing.

The rat’s hippocampus is differentially involved in working (short-term) and reference (long-term) memory (Olton and Papas, 1979) and may be affected by anxiety (Deacon and Rawlins, 2005). When considering the integration of OS and behavior, previous studies have reported positive correlations between hippocampus TBARS and RME (Gamoh et al., 2001; Haque et al., 2006; R^2^ = 0.86 and 0.52, respectively). Additionally, plasma TBARS were positively correlated with WME (R^2^ = 0.62; Haque et al., 2006). Serum TBARS had similar correlations with maze performance parameters in our study, indicating the impact of OS on maze learning performance and working memory; however, serum TBARS and WME were not different among rats in different dietary treatments. Additionally, OS markers were not correlated with anxiety behaviors in the current study. While OS has been tied to an increase in rodent anxious behaviors (Vollert et al., 2011), these studies assess anxiety via the open field and light-dark exploration tests. Some OS markers appear to be associated with maze learning performance; however, future studies should assess OS marker influence on anxiety directly through more accurate testing via open field or light-dark exploration tests.

## CONCLUSIONS

It was hypothesized that rats fed higher concentrations of dietary vitamin E would have suppressed OS markers, superior maze learning performance, and fewer anxiety behaviors. Contradictory to our hypothesis, the rats fed the highest concentration of vitamin E had reduced SOD and increased PC concentrations. Further contrary to our hypothesis, similar performance in maze learning performance was observed among rats fed varying vitamin E intakes. However, highest dietary vitamin E concentrations appeared to reduce rat anxiety behaviors. This supported our hypothesis but requires further investigation. Plasma TBARS, while not different across rats on different dietary treatments, were correlated with higher WME, indicating association of lipid peroxidation and working, short term memory.

In conclusion, although the higher concentrations of dietary vitamin E resulted in possible reductions in anxiety behaviors, concentrations greater than 90-ppm did not alter rat maze learning performance and were correlated with greater OS levels compared to rats fed a concentration of dietary vitamin E closer to published requirements. These results suggest higher concentrations of dietary vitamin E are not beneficial for OS mitigation or for rat maze learning performance.
